# Design and methodology of the Shanghai child and adolescent large‐scale eye study (SCALE)

**DOI:** 10.1111/ceo.13065

**Published:** 2017-10-27

**Authors:** Xiangui He, Rong Zhao, Padmaja Sankaridurg, Jianfeng Zhu, Thomas Naduvilath, Yingyan Ma, Lina Lu, Minzhi Lv, Earl L Smith, Serge Resnikoff, Kovin Naidoo, Haidong Zou, Xun Xu

**Affiliations:** ^1^ Department of Preventative Ophthalmology Shanghai Eye Disease Prevention and Treatment Center, Shanghai Eye Hospital Shanghai China; ^2^ Department of Ophthalmology, Shanghai General Hospital Shanghai Jiao Tong University Shanghai China; ^3^ Brien Holden Vision Institute Sydney New South Wales Australia; ^4^ School of Optometry and Vision Science University of New South Wales Sydney New South Wales Australia; ^5^ College of Optometry University of Houston Houston Texas USA

**Keywords:** children, high myopia, myopia, study design, vision impairment

## Abstract

**Importance:**

Nearly half of children suffering vision impairment reside in China with myopia accounting for the vast majority.

**Background:**

To describe the design and methodology of the Shanghai Child and Adolescent Large‐scale Eye Study (SCALE).

**Design:**

The SCALE was a city wide, school‐based, prospective survey.

**Participants:**

Children and adolescents aged 4–14 years from kindergarten (middle and senior), primary schools and junior high schools of all 17 districts and counties of the city of Shanghai, China were examined in 2012–2013.

**Methods:**

Each enrolled child underwent vision assessment (distance visual acuity; uncorrected and with corrective device if worn) and their parent/carer completed a questionnaire designed to elicit risk factors associated with myopia. Additionally, non‐cycloplegic autorefraction and ocular axial length was measured in a subset of the larger sample.

**Main Outcome Measures:**

Prevalence and the associated factors of vision impairment, myopia and high myopia in Shanghai.

**Results:**

In 2012–2013, a total of 910 245 of the eligible 1 196 763 children and adolescents identified from census (76%, mean age 9.0 ± 2.7 years [4–14 years]) were enrolled with visual acuity screened in the city of Shanghai. Of these, 610 952 children (67% of the entire sample) underwent non‐cycloplegic autorefraction and 219 188 (24% of the entire sample) had both non‐cycloplegic autorefraction and axial length measurements.

**Conclusions and Relevance:**

The study results will provide insights on the burden of vision impairment, myopia and high myopia in children and adolescents in a metropolitan area of China, and contribute to the policies and strategies to address and limit the burden.

## Introduction

Vision impairment in children can compromise their educational, social and psychological development, and may subsequently affect their employment and social economic status in adult life.^1^ Uncorrected refractive error is a common cause of vision impairment in children,^2^ with myopia being the most frequently seen refractive error, especially in East and Southeast Asian countries,^3^ such as Singapore, Japan, South Korea, Hong Kong, Taiwan, and parts of mainland China.^4^, ^5^, ^6^, ^7^, ^8^, ^9^, ^10^, ^11^, ^12^, ^13^, ^14^, ^15^ Elsewhere in the world as in North America, Australia, Europe and Middle East, the prevalence of myopia is also on the rise but at a slower pace.^16^, ^17^, ^18^, ^19^ As a consequence, the prevalence of high myopia is also on the rise. It is said that by 2050, nearly 10% of the global population will have high myopia with the prevalence being much higher in Asian countries.^20^ And indeed, prevalence of high myopia is already high in many parts of Asia^9^, ^10^, ^11^, ^21^, ^22^ and is one of the leading causes of vision impairment and blindness in adults.^23^, ^24^, ^25^ Thus there is an urgent need to understand the prevalence of vision impairment, myopia and high myopia and to set public health policies and strategies to manage the burden.^26^

Observations from a number of population‐based studies in China,^9^, ^10^, ^11^, ^12^, ^13^, ^14^, ^15^, ^27^, ^28^, ^29^, ^30^, ^31^, ^32^ show that myopia prevalence varies based on location and age. For example, in children aged 10 years myopia prevalence can range anywhere from 9% to 53%.^9^, ^12^, ^13^, ^15^, ^29^, ^30^ Also, myopia is increasingly being seen in younger, preschool children but very few studies have documented the prevalence of myopia in these populations. These limit our ability to understand the burden of vision impairment and myopia across the population to be able to institute required solutions.

The Shanghai Child and Adolescent Large‐scale Eye Study (SCALE) was a citywide, prospective, school‐based survey undertaken to address the ocular health needs of children aged 4–14 years old for the entire city of Shanghai. The primary objective of the study was to determine the prevalence and reasons for vision impairment as well as rate of spectacle lens usage. Secondary aims included estimating the prevalence of myopia and high myopia and determining risk factors associated with myopia and high myopia. Furthermore, the study aims to follow a subset of the larger cohort over a period of time to monitor the changes of prevalence and related factors. The results of the study will provide direction and strategies for public health services to manage the burden of vision impairment in children for example, determination of appropriate primary eye care services of children and strategies for prompt referrals, addressal and management of children with vision impairment and refractive errors.

## Methods

### Study population and sampling process

The SCALE was a prospective, school‐based study conducted from 2012 to 2013. The study aimed to include all children aged 4 to 14 years from the kindergarten (middle and senior) classes, primary schools and junior high schools in the 17 districts and counties of Shanghai. SCALE was funded by Shanghai Three‐year Action Plan for Public Health Systems Construction (2011–2013). The study protocol was approved by the Institutional Ethics Committee of Shanghai General Hospital, Shanghai Jiaotong University and follows the tenets of the Declaration of Helsinki for experimentation on humans. Parents or guardians or carers of all children in Shanghai were contacted and informed of the study purpose and procedures and written informed consent was obtained.

The city of Shanghai, covering an area of nearly 6340.5 km^2^, with a permanent resident population of 23 026 600 (census 2010) is the largest city by population in China. In 2012, according to data from Shanghai Bureau of Statistics, there were 1 196 763 children in the age group of 4–14 years old. The net attendance rate of children from this age group into kindergarten middle and senior classes was 98% and into primary and junior high schools was 99.9%. Each child enrolled in the study was required to undergo (i) distance visual acuity (including uncorrected visual acuity and visual acuity with corrective devices if any) and (ii) a questionnaire. Further examination included non‐cycloplegic refraction and axial length. Commencing 2015, a continuous investigation was initiated annually covering 30% of the entire eligible population, stratifying the participants by age and location (district and school).

### Study committee

**Figure 1:**
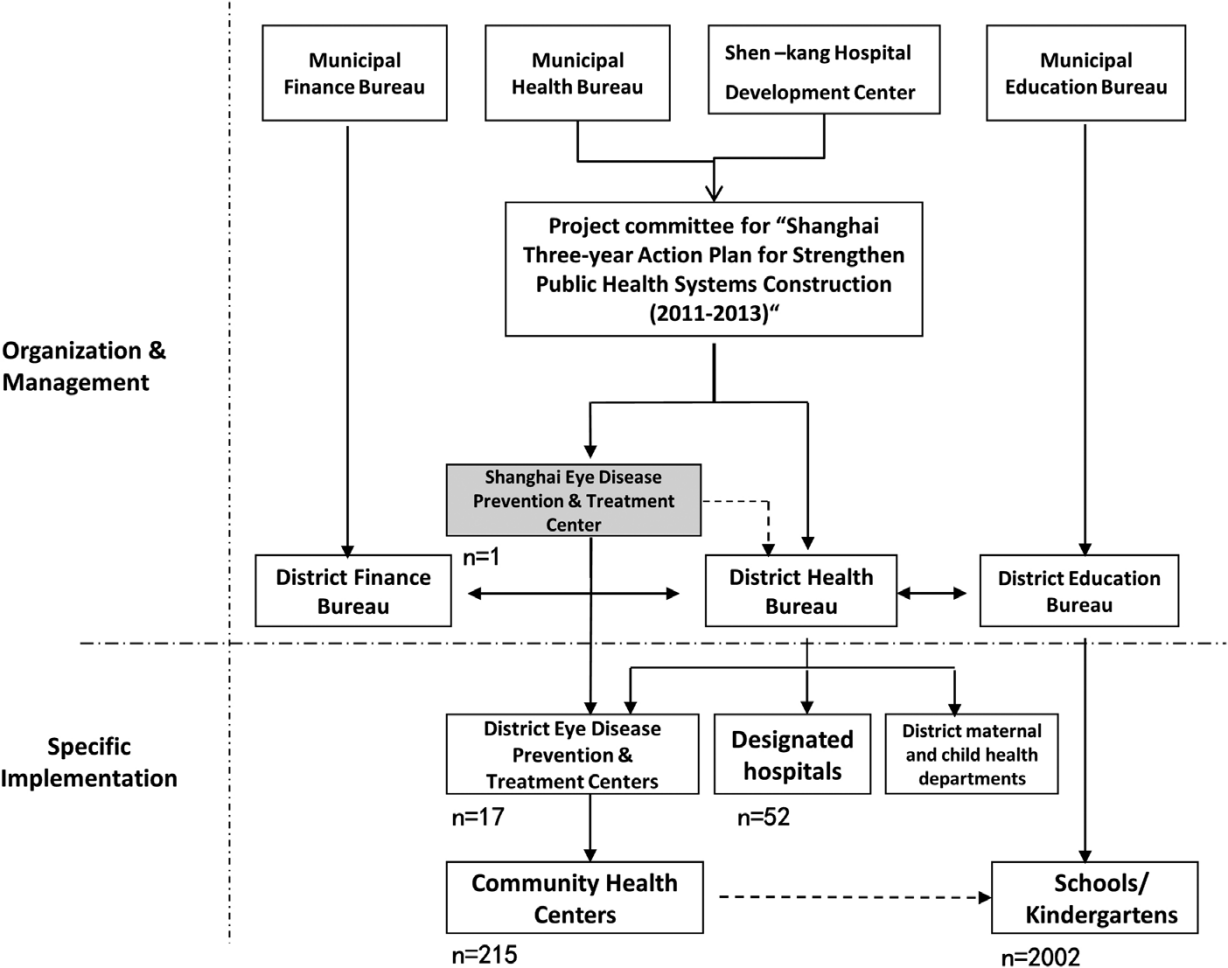
The framework of the organization, management and implementation in the SCALE study.

Led by Shanghai Municipal Health, Education and Finance Bureaus, a study committee was established to oversee, coordinate and administer the study. The study committee set an office at Shanghai Eye Disease Prevention and Treatment Center for day‐to‐day management and implementation of the study. In addition to the main administering centre, each district (county) also had established offices and a district (county) level project group that involved health and medical institutions and schools (district‐level eye disease control and prevention branch centres, maternal and child health‐care institutions, community health service centres, technical support hospitals, kindergartens, primary and secondary schools) (Fig. 1).

The study committee also organized experts to conduct field supervision and quality control at least once per year during the study period in each district (county). The district‐level project group organized field supervision and quality control across all study sites, including all kindergartens and schools examined, at the beginning and the end of the investigation.

### Study recruitment and data collection procedures

Prior to commencement, information about the study was disseminated to all kindergartens, primary and secondary schools and communities. The purpose and the study procedure was extensively publicized via newspapers, radio broadcasts, television, posters, electronic rolling screens, meetings with parents, text messages, open letters and themed activities. At the district level, all schools and kindergartens were required to be enrolled and all of the selected schools and kindergartens participated in the study. Each of the teams (a total of 17 teams, one per district) that conducted the screenings comprised of two public health doctors (to conduct visual acuity examination), two optometrists (for performing auto‐refraction and axial length measurements), one general coordinator/team leader (supervision of site) and two auxiliary personnel (stand‐by to assist in examination and inspect collected data). The team were recruited from staff engaged in primary eye health care for children, were trained and many of them had previously been involved in a pilot screening program conducted from 2007 to 2009 (Shanghai Three‐year Action Plan for Public Health Systems Construction).

Prior to the team visiting the school, printed copies of Brochure of Children's Refraction Development (hereinafter referred to as the *Brochure*, translated copy – Supporting information) were distributed to parents through schools and the signed informed consents were returned by parents to the school. The school returned the signed informed consent to the community health service centres.

**Figure 2:**
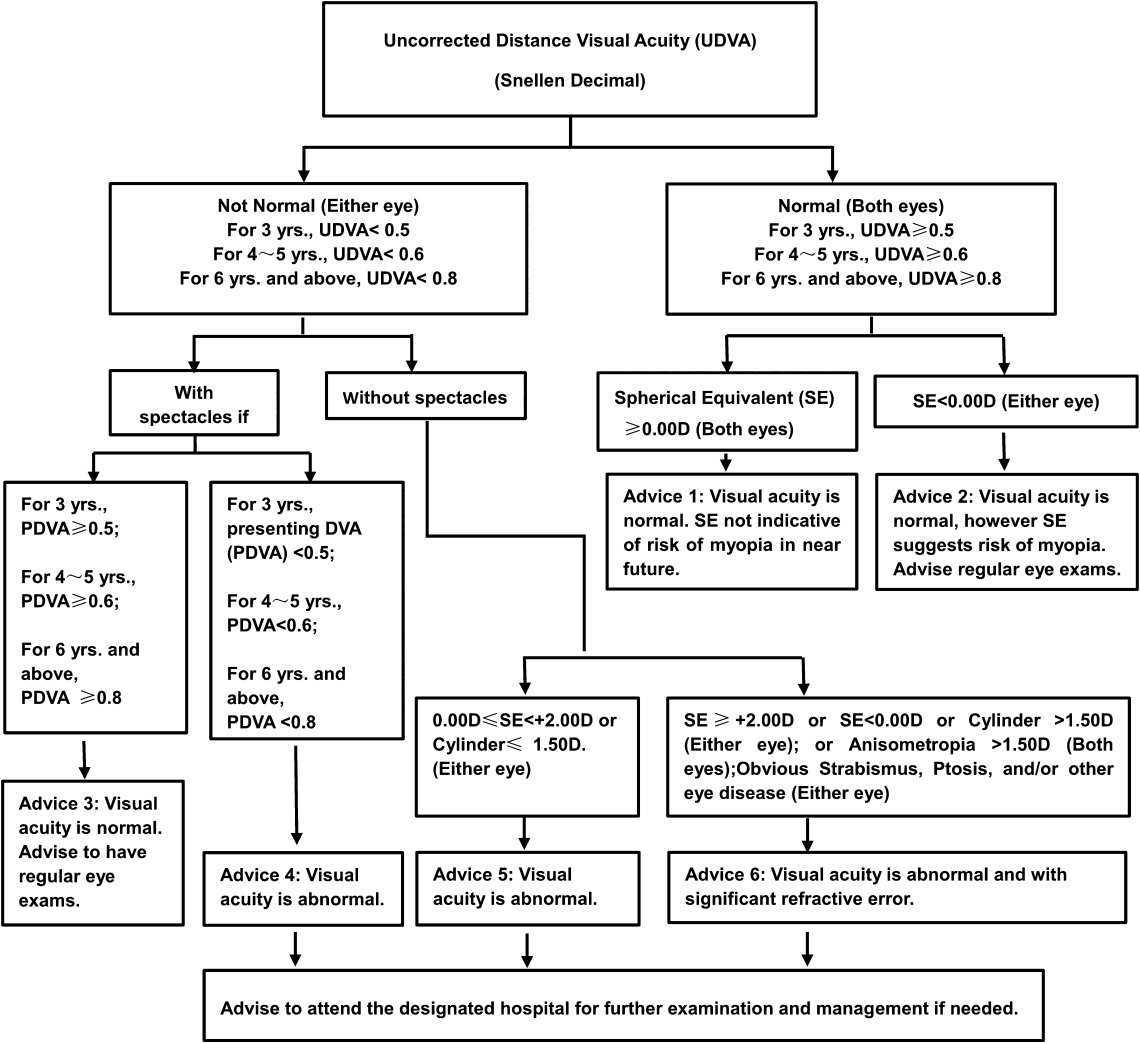
Criteria for referral to fixed‐point hospitals in the SCALE study (PDVA, presenting distance visual acuity; SE, spherical equivalent; UDVA, uncorrected distance visual acuity).

The process of data collection was as follows: (i) contact kindergartens/schools and organize the investigation days/schedule for screening; (ii) choose suitable examination site (indoor site which was usually the classroom or the sports hall); (iii) perform the examination and data collection and (iv) provide feedback of the examination to the children and parents. If visual acuity or refraction was not deemed to be normal (as outlined in Fig. 2), a referral was issued for children to travel to fixed‐point hospitals accompanied by parents to have re‐examination of visual acuity, cycloplegic and subjective refraction conducted by ophthalmologists or optometrists with experience in diagnosis and treatment of ocular conditions. All the information related to the study was recorded in printed copies of the *Brochure*. The section of the brochure requiring demographic details of the child was completed by the school, the section with the questionnaire was filled in by the parents and the data related to screening was filled in by the study team. Finally, the data recorded in the *Brochure* was entered into an online database by doctors in the community.

To assess the reliability of observers conducting the study procedures, correlation of data collected by the study team was compared to that of the data collected by the supervisor (standard). From each district, a study team was randomly selected and reliability of visual acuity test, auto‐refraction, and axial length measurements between the supervisor and the members of the team was assessed. Data was collected on the right eyes of 148 children. The mean paired difference between observer and the standard was 0.01 (95%CI: 0.00 ~ 0.02; *P* = 0.04) for uncorrected visual acuity and its intra‐class correlation was 0.93 (*P* < 0.001). Similarly, the mean paired difference for spherical equivalent was 0.04D (95%CI: −0.04 ~ +0.12D, *P* = 0.316) and its intra‐class correlation was 0.97 (*P* < 0.001). For axial length, the paired difference was −0.01 mm (95%CI: −0.03 ~ 0.001; *P* = 0.072) and its intra‐class correlation was 0.99 (*P* < 0.001).

#### *Questionnaire*

Prior to the examination, parents or carers together with children were required to complete a previously validated questionnaire that included questions on behavioural patterns of the child and was designed to elicit known risk factors for myopia.^33^ The questionnaire consisted of three sections. Section 1 was directed to factors that could impact eye health such as birth weight, duration of pregnancy, parental myopia and the age at which the child started to write with a pencil, Section 2 was directed to weekly time spent on activities such as near work, entertainment, outdoor activities, sports, extracurricular activities and art; and Section 3 considered whether the child took (i) breaks after continuous use of eyes for 30–40 min, (ii) if distance to books or digital tablets was less than 30 cm during reading or playing, (iii) if during distance viewing, distance to TV was less than 2 m and/or if distance to computer was less than 50 cm and (iv) whether the child adopted an unhealthy posture (e.g. head bent over the desk, if nib‐fingertip distance less than 1 inch whilst holding a pencil). The responses to Section 3 were coded as ‘never’, ‘occasionally’, ‘often’, respectively. The questionnaire is presented in the Supporting information.

#### *Distance visual acuity examination*

Uncorrected distance visual acuity was measured using a standard logarithmic visual acuity E chart (adhering to the National Standard of People's Republic of China, GB 11533–1989) mounted on an illuminated cabinet with a luminance of 80–320 cd/m^2^. The procedure was in accordance with the International criteria for recording visual acuity (Table 1 **)**.^34^, ^35^ Both unaided and presenting (with individual's own prescription where applicable) visual acuity were recorded and if a prescription was worn, the power of the prescription was measured. Visual acuity (VA) examination was conducted at a distance of 5 m from the chart and monocular VAs were recorded. Children found to be uncooperative were urged and guided by the teachers to complete the examination and if they were still uncooperative, they were re‐examined once the remainder of the children completed their examination. If they continued to be uncooperative, the reason for the lack of data was recorded.

**Table 1:** Conversion of the five‐grade notation to Snellen and log MAR

Five‐grade notation	Snellen (fraction)	Snellen (decimal)	logMAR
3.0	5/500	0.01	2
4.0	5/50	0.1	1
4.1	5/40	0.12	0.9
4.2	5/32	0.15	0.8
4.3	5/25	0.2	0.7
4.4	5/20	0.25	0.6
4.5	5/16	0.3	0.5
4.6	5/13	0.4	0.4
4.7	5/10	0.5	0.3
4.8	5/8	0.6	0.2
4.9	5/6	0.8	0.1
5.0	5/5	1.0	0.0
5.1	5/4	1.2	−0.1
5.2	5/3	1.5	−0.2
5.3	5/2.5	2.0	−0.3

#### *Auto‐refraction and axial length measurement*

**Figure 3:**
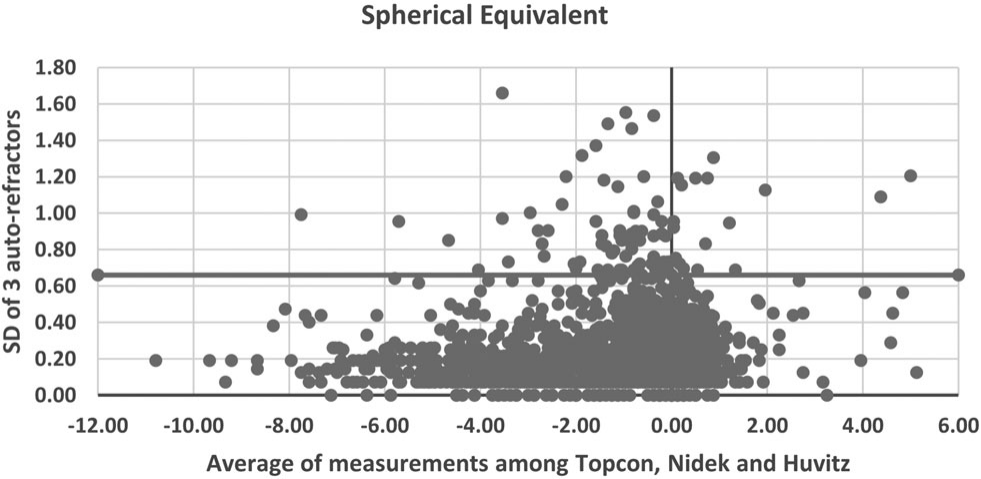
Standard deviation of the differences for the spherical equivalent refractive error between three auto‐refractors in the SCALE study (the three auto‐refractors: Topcon KR‐8900, Tokyo, Japan; Nidek AR‐330A, Nagoya, Japan; HUVITZ HRK‐7000A, Gemjeong‐dong, South Korea).

**Figure 4:**
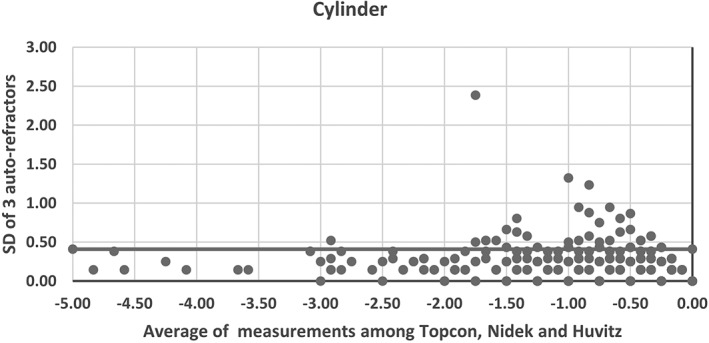
Standard deviation of the differences for the cylinder refractive error between three auto‐refractors in the SCALE study (the three auto‐refractors: Topcon KR‐8900, Tokyo, Japan; Nidek AR‐330A, Nagoya, Japan; HUVITZ HRK‐7000A, Gemjeong‐dong, South Korea).

Only certain numbers of schools were selected for assessment of non‐cycloplegic autorefraction and axial length measurements. The procedure was as follows: all schools in each of the districts were stratified to either key schools or ordinary schools based on their geographical location and economic criteria. Required number of samples, that is, schools were then randomly selected from these two stratums. Non‐cycloplegic auto‐refraction and corneal curvature measurements were conducted using either the Topcon KR‐8900 (Tokyo, Japan), Nidek AR‐330A (Nagoya, Japan) or HUVITZ HRK‐7000A (Gemjeong‐dong, South Korea) auto‐refractors.^36^, ^37^ Each eye was measured three times. If the difference between spherical equivalent refraction values of any two examinations was 0.50D or greater, an additional measurement was taken. If valid measurement was not possible, this was recorded in the child's *Brochure*. An assessment of the inter‐instrument correlation between the three auto‐refractors (Topcon, Nidek and Huvitz) was conducted using data from eyes of 1760 children (one kindergarten, two primary schools, four secondary schools of two districts). Each child was measured on the three devices in a random manner. A repeated measure ANOVA was used to assess the magnitude of spherical equivalent refractive errors and cylindrical refractive errors. The mean differences for spherical equivalent refractive error between the Topcon and Nidek, the Topcon and Huvitz, and the Nidek and Huvitz were 0.07D (95%CI: 0.05 to 0.09, 0.02D (95%CI: −0.00 to 0.04) and −0.05D (95%CI: −0.07 to −0.03) and for cylindrical refractive error were −0.05D (95%CI:−0.06 to −0.04), 0.01D (95%CI:−0.00 to 0.03) and 0.07D(95%CI: 0.05 to 0.08), respectively (*P* < 0.01). Figures 3 and 4 show the inter‐instrument repeatability for spherical equivalent and cylindrical power respectively, where the *X*‐axis is the average of the three instruments and the *Y*‐axis is the standard deviation of the three instrument measurements. For spherical equivalent, 90% of all participants have their inter‐instrument SD within 0.5D. There was no significant trend of inter‐instrument SD with spherical equivalent (*r* = 0.14). For cylindrical power, 98% of all participants have their inter‐instrument SD within 0.5D. The coefficient of reproducibility for spherical equivalent was 0.66 and 0.41 for cylindrical power. The intra‐class correlation of these three instruments was 0.97 (*P* < 0.001) for spherical equivalent and 0.86 (*P* < 0.001) for cylindrical power. It is likely that some participants showing an increased measurement error was due to variation in accommodative effort of eyes as only non‐cycloplegic measurements were conducted in this study. Also, the variation may be higher with hyperopic eyes and younger age groups. An IOLMASTER (version 5.02, Carl Zeiss Meditec, Jena, Germany) was used to measure the axial length in a further sub‐sample with three measurements per eye. The eye was re‐measured if the difference between two measurements was 0.02 mm or greater, the signal‐to‐noise ratio (SNR) was 2.0 or less, or there were two wave crests. Calibration of the instruments was performed at the start of each examination day.

#### *Feedback to parents/carers*

All the information collected with the survey and the examination was transferred to the *Brochure* and was sent to the child's parents/carer by the school health‐care teacher and included referrals, if any, to the fixed‐point hospital. A referral to the fixed‐point hospital was not compulsory. If the child attended the fixed‐point hospital, results of any examination/assessments conducted at the fixed‐point hospital were also recorded in the *Brochure*.

### Data analysis

Data from the screening examination and the questionnaire were entered by qualified community public health doctors into a dedicated database developed using Microsoft SQL (Version 2005, Microsoft Corporation, Redmond, WA, USA). The online database could be accessed at multiple points within the various jurisdictions. The system used a unique identification card number for each child. The database had inbuilt checks for variables. A unique serial number was generated for each child that was used while verifying and analysing the data to mask the identity of each child. At intervals, data quality was assessed by auditing the data entry variables against the original entries in the *Brochure*. One class from each district was randomly selected for assessing the quality of data entry. If the differences between the *Brochure* and the electronic records were more than 1% of the content, all of the brochures of the whole class students would be checked and any error found would be corrected, then records from another class was checked and the process continued until the difference was less than 1%.

Data verification and analysis will be performed using SAS 9.3 (SAS Institute, Cary, NC, USA) and R3.2.0 (Vienna, Austria) by two statisticians, respectively. Incorrect entries of the database were checked by abnormalities in the distributions of the variables and any error found would be corrected if available otherwise treated as missing. The prevalence of vision impairment (VI) will be assessed based on presenting VA in the better eye as defined by WHO categorization^38^, ^39^ wherein no VI was defined as VA ≥ 6/12, mild VI was VA <6/12 but ≥6/18, moderate VI was VA <6/18 but ≥6/60, severe VI was VA <6/60 but ≥3/60 and blindness was defined as VA <3/60. The prevalence of VI due to myopia and the spectacle usage will also be calculated. A subset of 6017 school children aged 4–14 years old will have both non‐cycloplegic and cycloplegic (with 1% cyclopentolate eye drops) refractions performed and a model developed to determine if eyes can be categorized as myopic, emmetropic or hyperopic based on non‐cycloplegic refraction. Eyes with spherical equivalent (SE) refractive error ≤ −1.0D will be categorized as myopic and those with SE ≤ −5.0D will be categorized as having high myopia. Further analysis on the prevalence of vision impairment, myopia and high myopia will be performed using appropriate statistical techniques.

## Results

### Baseline information

The study was conducted from January 2012 to December 2013. Of the 1 196 763 children identified as eligible based on census, a total of 910 245 children were enrolled providing an enrolment rate of 76.06%. Table 2 provides details of the number of children enrolled for each age from 4 to 14 and Table 3 provides details of children enrolled from each district (total of 17 districts). The mean age of the enrolled population was 9.04 ± 2.78 yrs. The difference showed statistically significance between eligible 1 196 763 children (9.13 ± 3.01 years) and enrolled 910 245 children because of the large sample size (*t* = 25.47, *P* < 0.001) but not obvious in absolute values. And there was no statistical significance in the distribution of gender between eligible and enrolled population (the proportion of boys were 53.3% *vs*. 53.3%, respectively, Pearson Chi‐square = 0.870, *P* = 0.351).

**Table 2:** Age and gender distribution of the enrolled children in the SCALE study

Age	Eligible children	Enrolled children (%)	Male no. (%)	Female no. (%)	No of children with non‐cycloplegic refraction (%)	No of children with axial length (%)
4	78 673	27 360 (34.8)	14 431 (52.7)	12 929 (47.3)	17 511 (64.0)	6968 (25.5)
5	79 954	73 826 (92.3)	39 051 (52.9)	34 775 (47.1)	51 600 (70.0)	20 188 (27.3)
6	126 376	91 044 (72.0)	48 290 (53.0)	42 754 (47.0)	60 690 (66.7)	26 299 (28.9)
7	118 568	113 140 (95.4)	60 691 (53.6)	52 449 (46.4)	74 481 (65.8)	33 004 (29.2)
8	122 202	113 392 (92.8)	61 203 (54.0)	52 189 (46.0)	74 992 (66.1)	31 835 (28.1)
9	119 508	106 997 (89.5)	57 316 (53.6)	49 681 (46.4)	72 604 (67.9)	29 984 (28.0)
10	121 579	90 577 (74.5)	48 414 (53.4)	42 163 (46.6)	62 252 (68.7)	25 873 (28.6)
11	108 830	87 351 (80.3)	46 418 (53.1)	40 933 (46.9)	60 585 (69.4)	22 038 (25.2)
12	110 197	74 329 (67.5)	39 346 (52.9)	34 983 (47.1)	49 467 (66.6)	9792 (13.2)
13	106 070	71 688 (67.6)	37 603 (52.4)	34 085 (47.6)	47 285 (66.0)	7382 (10.3)
14	104 806	60 541 (57.8)	31 989 (52.8)	28 552 (47.2)	39 485 (65.2)	5825 (9.6)
Total	1 196 763	910 245 (76.1)	484 752 (53.3)	425 493 (46.7)	610 952 (67.12)	219 188 (24.08)

SCALE, Shanghai Child and Adolescent Large‐scale Eye Study.

**Table 3:** District distribution of the enrolled children in the SCALE study

District	Eligible children	Enrolled children	Percent enrolled (%)	Mean age(year) (Mean ± SD)	Male:female (%)
Huangpu	38 786	35 862	92.46	9.56 ± 2.89	52.8: 47.2
Xuhui	68 326	29 125	42.63	8.48 ± 1.84	53.0: 47.0
Changning	42 202	38 883	92.14	9.06 ± 2.86	52.2: 47.8
Jingan	26 235	23 818	90.79	8.55 ± 3.21	52.6: 47.4
Putuo	63 554	56 968	89.64	8.80 ± 2.93	53.2: 46.8
Zhabei	39 872	37 208	93.32	9.05 ± 2.99	53.3: 46.7
Hongkou	46 208	10 896	23.58	10.47 ± 2.53	50.8: 49.2
Yangpu	62 840	59 567	94.79	8.98 ± 3.02	52.6: 47.4
Pudong	266 243	140 935	52.93	9.02 ± 2.35	53.4: 46.6
Minhang	97 391	81 532	83.72	8.76 ± 2.81	53.4: 46.6
Jinshan	48 385	40 779	84.28	9.02 ± 2.84	52.8: 47.2
Songjiang	64 859	55 952	86.27	8.89 ± 2.74	53.8: 46.2
Qingpu	65 373	58 886	90.08	9.04 ± 2.86	54.1: 45.9
Fengxian	69 641	57 605	82.72	9.29 ± 2.73	54.2: 45.2
Baoshan	94 793	89 885	94.82	9.12 ± 2.75	53.7: 46.3
Chongming	38 324	33 030	86.19	9.19 ± 2.97	51.6: 48.4
Jiading	63 731	59 314	93.07	9.28 ± 2.92	53.5: 46.5
Total	1 196 763	910 245	76.06	9.04 ± 2.78	53.3: 46.7

Of the 910 245 children, 610 952 children (67% of the sample) underwent non‐cycloplegic auto‐refraction and among them 219 188 (24% of the sample) had both non‐cycloplegic and axial length measurements. There was no significant difference in the distribution of age and gender between children with and without non‐cycloplegic refraction (mean age 9.03 ± 2.77 *vs*. 9.05 ± 2.80 years, the proportion of boys 53.2% *vs*. 53.3%, respectively).

## Discussion

Data from the SCALE study will not only provide information on the prevalence of vision impairment in children and adolescents aged 4 to 14 for the entire city of Shanghai but is also likely to aid in understanding the prevalence of myopia, high myopia and related risk factors. In addition to understanding the magnitude of the problem, these data will provide valuable insights to predicting the burden of myopia, high myopia and vision impairment. Furthermore, our plan to conduct a continuous assessment of a subset of the children within Shanghai will help monitor the changes, if any, in the prevalence of vision impairment and myopia.

Although SCALE considered enrolling and screening every eligible child, in designing and conducting the study, a number of practical difficulties were encountered that made that goal unachievable and finally 76% of all eligible children were screened. One of the reasons for not achieving 100% enrolment was lack of resources from districts and counties to conduct the screening. For example, in three of the 17 districts (Table 3, Xuhui, Hongkou and Pudong) less than 53% of the eligible children were enrolled due to lack of resources. In addition, the percent of enrolled children was less in the young (difficulties due to cognitive ability and cooperation with visual acuity testing) and in children aged 12 years and above (moved to high schools). In spite of these limitations, the large sample size and the fact that we had considered special populations (the study conducted screening in the single school for vision impaired and blind) indicate that the results are generalizable to the population of Shanghai with respect to the prevalence of vision impairment.

Prevalence of vision impairment was calculated using the WHO definition of presenting VA in the better eye.^38^, ^39^ Unfortunately, best corrected VA was not measured due to lack of human resources and may therefore limit our understanding of the level of correctable vision impairment in the population. However, we intend to determine the prevalence of uncorrected vision impairment using uncorrected VA and the rate of spectacle lens usage to estimate the extent of refractive correction coverage and the need of eye care services. Also, other epidemiological studies from China have reported the proportion of the uncorrectable vision impairment (best corrected VA in better eye were lower than 20/32) to be approximately 1–3% in 4–18 years old children and about 8% in 3–10 years old children whose uncorrected VA in better eye were lower than 20/32. 9, 12, 13 Also, given the strength of the sample size, a high degree of confidence can be placed in the association of risk factors to vision impairment. An additional strength of the study is that since the local health authorities have already engaged and embraced the study, designing and implementing strategies to reduce the burden by relying on their skills and knowledge with respect to managing large‐scale community‐based programs becomes invaluable.

Since Shanghai has a high myopia prevalence similar to other Asian countries,^10^, ^12^, ^40^ much of the vision impairment is likely to be due to myopia. In this respect, a limitation of the current study is that the refractive status of the children was assessed without cycloplegia and therefore likely to overestimate the prevalence of myopia.^41^, ^42^ Considerable effort and resources are needed to administer cycloplegia in children and we lacked the resources to administer cycloplegia in the current protocol. In addition, in this region, there exists a significant parental resistance for dilatation/cycloplegia and it would not have been feasible to conduct the study within the timeframe and resources allocated to the project if cycloplegia were required. However, if the vision impairment was the result of a refractive error such as myopia, it is likely that the refractive error will generally be of a high magnitude. In this respect it has been found that the difference between non‐cycloplegic and cycloplegic refractive error is much higher in eyes with high hyperopia, low refractive errors and emmetropia and less in myopic errors of moderate to high magnitude. Since distance vision impairment is more likely with higher levels of myopia, non‐cycloplegic measurements may still be of value in profiling the reasons for vision impairment and estimating the prevalence of myopia and high myopia. Also, uncorrected visual acuity combined with non‐cycloplegic auto‐refraction or axial length to corneal radius of curvature ratio was found to result in higher sensitivity and specificity for diagnosing myopia.^43^, ^44^ We plan to utilize some of these techniques/procedures to profile the prevalence of myopia in the population.

There are other limiting factors. Adolescents (>14 years) are far more likely to be affected by vision impairment due to the higher prevalence of myopia and high myopia in this age group, but were not considered in the study as it was not feasible to access them at high schools. Also, the prevalence of vision impairment, myopia and high myopia in children as estimated in the current study may be not extrapolated to other regions due to differences in exposure to urban environmental factors, access to resources etc.

In summary, despite its limitations, SCALE is the largest study to have considered the prevalence of vision impairment, myopia and high myopia in children and adolescent population aged 4 to 14 in Shanghai, China. It is hoped that the study will provide insights that prove valuable for planning of public health interventions to address the burden of vision impairment, myopia and high myopia.

## Supporting information


**Data S1.** Archives Brochure of Children's Refractive Error Development in Shanghai.
