# Supplementary material for: Design and methodology of the Shanghai child and adolescent large‐scale eye study (SCALE)
Source: Clin Exp Ophthalmol. 2017 Oct 27;46(4):329–38. doi: 10.1111/ceo.13065 (PMC6032906; doi:10.1111/ceo.13065)
Supplement: Supplementary file 1 — Data S1. Archives Brochure of Children's Refractive Error Development in Shanghai. [file CEO-46-329-s001.pdf]

Shanghai Three-year Action Plan for Public Health Systems Construction (2011-2013)

Archives Brochure of Children's Refractive Error Development in Shanghai

Archives number (ID number)□□□□□□□□□□□□□□□□□□

Name:________Date of Birth:____Year____Month ____Day

Gender:________Ethnicity:_______Native Place: _________

School:________School's Location:______District (County) ______

Shanghai Municipal Health Bureau
Shanghai Municipal Education Commission

Instruction for “Archives Brochure of Children’s Refractive Error Development”

Dear parents

In recent years, the rate of myopia in children was found to be particularly high in China, with onset in young children and developing to high myopia. It’s well known that myopia has detrimental effects on health of children and adolescents, leading to limited attention, decline of distant vision and fine work ability. The greater the degree of myopia, the greater the chances of developing complications such as macular degeneration and retinal detachment which can lead to visual impairment and blindness.

Recently, a people-benefit project named “Comprehensive Prevention and Control of Myopia in Children---Establishing Children’s Individual Ocular Refractive Development Archives” was launched by the Shanghai government with emphasis on the prevention and control of myopia in children. In this project, the refractive development of eyes of each child is recorded in this brochure and input into a database and established for all students from middle and senior grades in kindergartens, primary and middle schools.

Evidence shows that monitoring visual acuity and refractive status of eye routinely and dynamically as well as referring to archives not only helps detect refractive errors such as myopia, hyperopia, astigmatism and other eye diseases early on, but also helps identify children at risk of becoming myopic before symptoms appear, thus reducing the incidence of myopia and also slow the progression of myopia.

This program is organized and implemented by Shanghai Eye Disease Prevention and Treatment Center, with support from Shanghai Municipal Health Bureau, Shanghai Municipal Education Commission, and district level of Health Bureau and Education Commission in all districts (counties). District’s (county’s) eye disease prevention and treatment centers organize the designated hospitals and the communities to conduct the screening at schools. Archives are established after regular examination of children’s visual acuity and refraction each time.

Dear parents,

Ocular health is directly related to child’s physical and mental health and future prospects. Please look through your child’s examination results carefully and adopt measures based on the doctor’s advice.

Special note:

1. The school screening results are for your reference only, and cannot be considered as the final diagnosis. If advised, please go to designated hospital for further mydriasis and optometry examination.
2. Cannot be duplicated or copied or considered to be legal.
3. All rights reserved by Shanghai Eye Disease Prevention and Treatment Center.

Flow of the Brochure Procedure

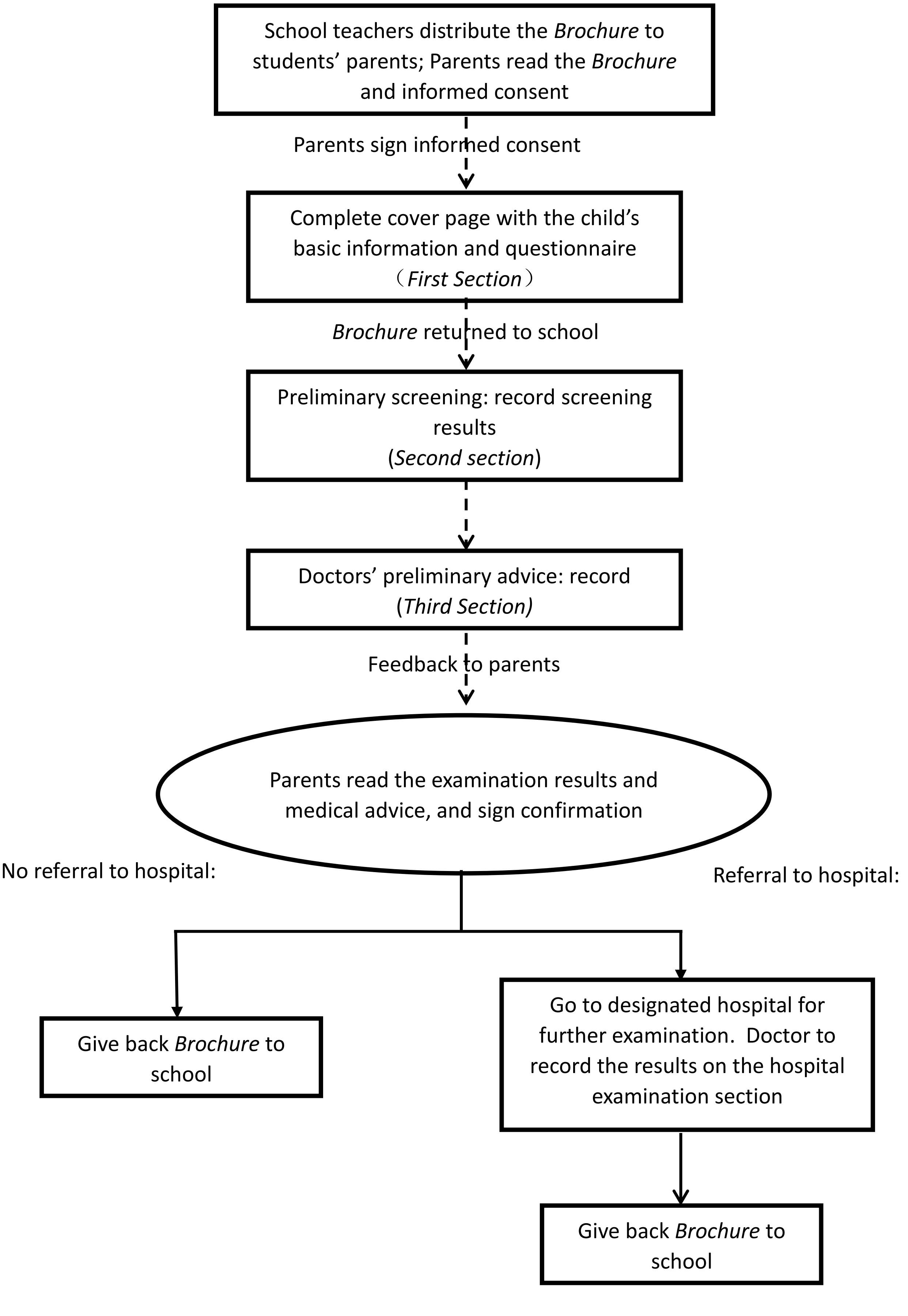

First Section: Myopia Questionnaire

In order to facilitate counseling and guidance for prevention and treatment of myopia, parents are asked to complete the following questionnaire.

1. Early influence factors (Parents fill the blank or tick ✓） in the box "☐")

1.1 Birth weight __________（g）

1.2 Premature birth: ☐No ☐Yes，_____weeks(Premature birth means babies born between 28 weeks and 37 weeks)

1.3 Myopia diopters of parents: father _____D, mother _____D, ☐ both without myopia

1.4 Age at which started to write with a pencil: _____ yeas old (write numbers, letters, Chinese characters etc. in the grid)

2. Duration of activity (please recall time spent by your child on near work, outdoor activities and sleep this semester)

Recent timetable	Week day (after school)	Weekend (Saturday, Sunday)
2.1 Finishing homework, reading books and magazines, etc. (Per day)	average____ hours___ minutes	average____ hours___ minutes
2.2 Entertainment (Per day)	average____ hours___ minutes	average____ hours___ minutes
2.3 Outdoor exercises and activities (Per day)	average____ hours___ minutes	average____ hours___ minutes
2.4 Wake-up time	Usually, Hour ____Minute_____	Usually, Hour ____Minute_____
2.5 Sleep time	Usually, Hour ____Minute_____	Usually, Hour ____Minute_____
2.6 Total amount of time for sport based activities (e.g. swimming, basketball, tennis, dance, etc.) ______hours_____ minutes per week.		
2.7 Total amount of time for extracurricular activities (e.g. English, mathematics, writing, etc.) _____hours ______minutes per week.		
2.8 Total amount of time for art activities (e.g. painting, calligraphy, chess, model, etc.) ______hours_____ minutes per week.		

3. Recent eye behavior（please recall the behaviour of your child on the following activities at home this semester, and tick the appropriate option（✓））

Habits	Often	Sometimes	Rarely
3.1 Rest after continual use of eyes for 30-40 minutes (including distance gazing, close eyes, exercises etc.)			
3.2 Distance from eyes to books or Ipad is less than 30 cm while reading or playing.			
3.3 Distance from TV sets or computer is less than 2m or 50cm while watching.			
3.4 Doing homework with an unhealthy posture, such as bending over the desk, tossing their heads, wrong pen holding gesture—nib-fingertip distance less than 1 inch.			

Second Section: School/Kindergarten Screening Results

Grade(Class): ______ Examination time :_______ year ______ month ______ day
Height___ （cm） Weight____(kg)

Please write using 5 points recording method

	Uncorrected Visual Acuity	Visual Acuity with Spectacles
Right Eye		
Left Eye		

(Visual Acuity with Spectacles means vision with children's own spectacles.)

	Sphere	Cylinder (Astigmatism)	Axis of Astigmatism
Right Eye			
Left Eye			

	Corneal Curvature (D)		Axial Length （mm）
	H	V	
Right Eye			
Left Eye			

Other special remarks:_ ____

Attach auto-refraction printed records

Notes：
1. Normal uncorrected visual acuity: 3 years old ≥4.7，4～5 years old ≥4.8，6 years old and above≥4.9
2. Visual acuity with spectacles means vision with children's own spectacles, for Primary and Middle School students it should be ≥4.9 except for amblyopia, tropia or other eye pathologies.
3. For auto-refraction, "Sphere" means degree of myopia or hyperopia; The negative sign "-"represents for myopic, while positive for hyperopic; "Cylinder" means degree of astigmatism; The axis of astigmatism represents the direction of corneal astigmatism. Only when there be astigmatism can there be axis.
4. This refraction is tested under non-cycloplegic conditions. The results are for reference only and cannot be used for diagnosis.

Medical advice on the third section

Third Section: School/Kindergarten Screening Result Feedback

Medical advice

□1.Uncorrected visual acuity is in normal range, and currently no evident risk factors for myopia onset.

□2. Visual acuity with spectacles is in normal range, please undergo uncorrected visual acuity and visual acuity with spectacles examination on a 6 monthly basis.

For the following, you are suggested to go to the designated hospitals for further consultation.

□ 3. Uncorrected visual acuity is in normal range, however there are risk factors for myopia onset. Advice ①pay strict attention to the ocular hygiene ②Please go to the designated hospital to check if child is myopic with cycloplegic and optometric examination.

□4. Uncorrected visual acuity is abnormal. Please go to the designated hospital for cycloplegic and optometric examination, clarify the diagnosis and ensure that appropriate intervention is used.

□5. Uncorrected visual acuity is abnormal and with significant refractive error. Please go to the designated hospital for diagnosis and treatment.

□6. Visual acuity with spectacles is abnormal. Please go to the designated hospital and check if spectacles need to be replaced.

Referral notice

Dear parents,

Based on your child's screening results, we suggest that you proceed for further examination

From_____Year_____Month_____Day ～_____Year_____Month_____Day, _____day of the week, Time _____～ Time_____

Please go to the designated hospitals with this Brochure. It is voluntary. The cost of examination is for free but costs of intervention if any requires payment.

Designated hospital: ××××

If you have any questions, please contact 12320 Public Health Hotline.

Parents Receipt

I have read, and understand my child's visual acuity and current feedback.

Parents' signature______________ Date___Year__Month__Day

For children need to be reviewed (medical advice 3-6), parents' consideration :

□Go to designated hospital for further examination;

□Go to other hospitals for further examination other than designated ones.

□The kid has been to see a doctor within last 3 months and no need for further exam.

□ I have no interest and will not participate in the next procedure.

Fourth Section: Hospital Examination

Visual Acuity

	Uncorrected Visual Acuity	Visual Acuity with Spectacles	Diopters of Spectacles		
			Sphere	Cylinder	Axis
Right Eye					
Left Eye					

(Visual Acuity with Spectacles means vision with children wearing their own spectacles; Diopters of Spectacles means diopters of their own habitual spectacles.)

Examination of Ophthalmology

Eye position: 1.□Normal 2.□Manifest strabismus, Degree___ 3.□Recessive strabismus, Degree ___

External eye： ____ Anterior segment： _____ Fundus：

IOP（NCT）： right eye _____mmHg left eye_____mmHg

Refraction Examinations:： Cycloplegia: □ Yes □ No

	Refraction			Corneal Curvature (D)		Axis Length (mm)	Subjective Refraction			
	Sphere	Cylinder	Axis	H	V		Sph ere	Cylinder	Axis	Best -corrected Visual Acuity
Right										
Left										

(the corneal curvature and axial length can be input from the screening results-second section)

Diagnosis(multiple choices)**:**

□1. Normal vision acuity (please fill in the following items about refractive development)

□2. Myopia □3. Hyperopia □4.Astigmatism □5.Anisometropia □6.Amblyopia □7 Strabismus

□8.Other

(Visual acuity normal range: 3 years old ≥4.7， 4～5 years old ≥4.8， 6 years old and above≥4.9)

Refractive development: □1.normal □2.deviate(for children with normal vision acuity only) (Normal reference range: cycloplegic-spherical equivalent: 3～5years old： +1.75～+2.00D；6～8 years old：+1.25～+1.50D; 9～12 years old:+0.75D～+1.00D;Astigmatism<0.75D)

Treatments (multiple choices):

Corrected with Lenses： □ Frame spectacles □Ortho-K lens □RGP □Other_______________

Medication： ____

Training： □ Accommodation function training □Acupuncture □Massage Others： ________

Doctor（seal）_________________ Hospital： ________________________

Examination Date： ___Year__Month__Day Parents' signature： _______________________

Back cover

Shanghai Public Health Hotline: 12320
Shanghai Eye Disease Prevention and Treatment Center Hotline: 021-62539696
Available hours: 13:00-16:00 Monday ~ Friday
www.shsyf.com/dangan

Shanghai Eye Disease Prevention and Treatment Center Production
